# Tubulin Inhibitor-Based Antibody-Drug Conjugates for Cancer Therapy

**DOI:** 10.3390/molecules22081281

**Published:** 2017-08-01

**Authors:** Hao Chen, Zongtao Lin, Kinsie E. Arnst, Duane D. Miller, Wei Li

**Affiliations:** Department of Pharmaceutical Sciences, University of Tennessee Health Science Center, 881 Madison Avenue, Room 561, Memphis, TN 38163, USA; hchen55@uthsc.edu (H.C.); zlin8@uthsc.edu (Z.L.); karnst@uthsc.edu (K.E.A.); dmiller@uthsc.edu (D.D.M.)

**Keywords:** antibody-drug conjugates, cytotoxic payloads, linker, monoclonal antibody, site-specific conjugation, tubulin inhibitors

## Abstract

Antibody-drug conjugates (ADCs) are a class of highly potent biopharmaceutical drugs generated by conjugating cytotoxic drugs with specific monoclonal antibodies through appropriate linkers. Specific antibodies used to guide potent warheads to tumor tissues can effectively reduce undesired side effects of the cytotoxic drugs. An in-depth understanding of antibodies, linkers, conjugation strategies, cytotoxic drugs, and their molecular targets has led to the successful development of several approved ADCs. These ADCs are powerful therapeutics for cancer treatment, enabling wider therapeutic windows, improved pharmacokinetic/pharmacodynamic properties, and enhanced efficacy. Since tubulin inhibitors are one of the most successful cytotoxic drugs in the ADC armamentarium, this review focuses on the progress in tubulin inhibitor-based ADCs, as well as lessons learned from the unsuccessful ADCs containing tubulin inhibitors. This review should be helpful to facilitate future development of new generations of tubulin inhibitor-based ADCs for cancer therapy.

## 1. Introduction

Cancer is one of the leading causes of death in the United States, accounting for about half a million deaths every year [1]. In the early days of cancer treatment, conventional chemotherapy dominated the field with an improvement in cancer-related mortality. Early chemotherapeutic agents included, but were not limited to, DNA-damaging agents (such as nitrogen mustard, anthracyclines, and cyclophosphamide), tubulin inhibitors (such as the vinca alkaloids vinblastine and vincristine, and taxane diterpenes like taxol), and antifolate agents (such as methotrexate, pemetrexed, proguanil, pyrimethamine, and trimethoprim). A significant proportion of cancer patients suffer from acquired resistance or relapse after long-term chemotherapy treatment. Moreover, these drugs usually have nonspecific toxicity which kills not only tumor cells, but also damages normal tissues and causes serious side effects that often limit their efficacy [2].

In contrast to chemotherapy, targeted therapy is considered a more efficacious and safer cancer treatment and has gained increasing interest in recent years. Targeted therapy blocks the rapidly dividing cancer cells by interfering with the specific targeted molecules needed for tumor growth [3]. The main categories of current targeted cancer therapy are small molecules (e.g., serine/threonine kinase inhibitors and tyrosine-kinase inhibitors), monoclonal antibodies (mAbs), and antibody-drug conjugates (ADCs). One of the most successful small-molecule targeted therapeutic agents is imatinib mesylate, a kinase inhibitor used for chronic myelogenous leukemia and gastrointestinal stromal tumor [4]. Other examples include erlotinib and bortezomib for treating non-small cell lung cancer (NSCLC) and multiple myeloma, respectively [5,6]. Another emerging targeted therapy is monoclonal antibody (mAb) therapy, also known as immunotherapy [7]. The mAbs are capable of binding specific tumor-associated antigens and inducing cancer cell death through antibody-dependent cell cytotoxicity, complement-dependent cytotoxicity, or interference with signaling pathways; thus they offer a tumor-targeted treatment approach [8,9]. Targeted mAb therapy is used to treat many cancers including non-Hodgkin’s lymphoma, colorectal cancer, head/neck cancer, and breast cancer. However, small-molecule or mAb targeted therapy alone often shows inadequate therapeutic activity due to its low cytotoxicity and weak penetration into solid tumors. An improved approach to overcome these limitations is to create ADCs.

**Figure 1:**
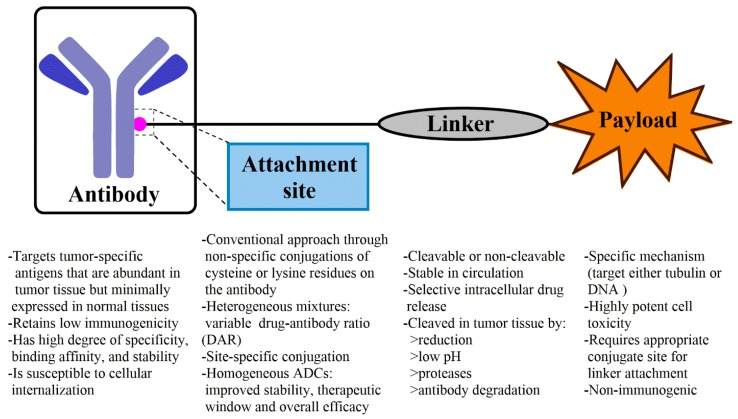
The general components of ADC. Selection principles of antibody, comparison between non-specific and specific conjugations, and descriptions on how the drug is released and its mechanism of action.

ADC is a complex molecule composed of a mAb conjugated with a highly potent cytotoxic drug (or payload) through an appropriate linker (Figure 1) [10,11]. An ADC is designed as targeted therapy, which targets and kills only cancer cells while sparing healthy cells. The development of ADCs faced a number of challenges in the early 1970s. The first set of ADCs, prepared by conjugating murine KS1/4 antibody with the cytotoxic drug methotrexate or the BR96 antibody conjugated with doxorubicin, were less active than their parent drugs [12,13,14]. The failure of early ADCs resulted from several factors: (a) the chimeric or murine antibodies used in ADCs could elicit immunogenic responses and cause fast clearance in circulation; (b) the cytotoxic drug was insufficiently potent, and further conjugation might lead to decreased potency; (c) inappropriate linkage and the poor selection of antigen could lead to the failure of an ADC.

Later ADCs achieved success after a better understanding of the basic requirements for their design. First, the application of humanized and human antibodies has greatly reduced the problems caused by immunogenicity. Second, internalizing antigens as tumor targets allowed the design of more functional antitumor antibodies. Third, understanding the mechanisms of action (protein uptake) of ADCs in mammalian cells helped design different types of linkers, which facilitated the release of payload inside the tumors. Finally, the discovery of new cytotoxic compounds with picomolar IC_50_ values further propelled the success of ADCs [15]. ADCs are now powerful therapeutics enabling wider therapeutic windows, improved pharmacokinetic/pharmacodynamic (PK/PD) profiles, and enhanced efficacies. The first ADC, gemtuzumab ozogamicin (Mylotarg^®^, Pfizer/Wyeth-Ayerst Laboratories, New York, NY, USA), was approved by the US Food and Drug Administration (FDA) in 2000 (later withdrawn from the market in 2010 due to toxicity concerns and no sufficient clinical benefit) for treating acute myeloid leukemia. It was followed by recent approvals of brentuximab vedotin (SGN-35, Adcetris^®^, Seattle Genetics, Inc., Bothell, WA, USA) in 2011 and ado-trastuzumab emtansine (T-DM1, Kadcyla^®^, Genentech, South San Francisco, CA, USA) in 2013. Encouraged by the success of these ADCs, many new ADCs have been developed. Currently, more than 55 ADCs are under various stages of clinical evaluation [16]. Since tubulin inhibitors are one of the most successful cytotoxic drugs incorporated in ADCs, this review focuses on the development of tubulin inhibitor-based ADCs.

## 2. General Considerations for ADC

### 2.1. Drug Release Mechanism of ADC

**Figure 2:**
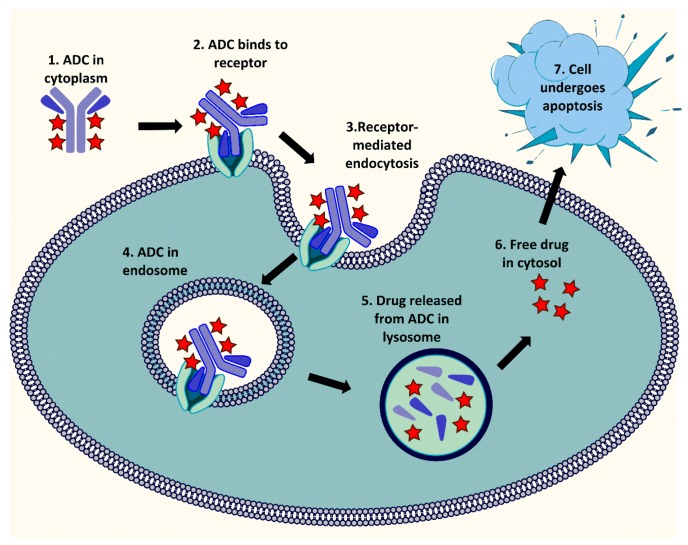
General mechanism of action of ADCs. The antibody of an ADC binds to tumor associated antigen. Upon binding, the ADC-antigen complex is internalized into the cancer cell. During the degradation of the complex, the released payload exerts its effects on the intracellular target leading to apoptosis.

ADCs are an important class of highly potent biopharmaceutical drugs designed as prodrugs for the treatment of cancers. The drug release mechanism of ADCs consists of several key steps. The ADC first binds to the tumor-associated antigen on the target cells. The resulting antigen-ADC complex is internalized by receptor-mediated endocytosis, and subsequently transferred to late endosome and lysosome where the cytotoxic drug is released either by cleavage of the linker or degradation of the antibody. Finally, the released payload affects the intracellular target leading to apoptosis and cell death (Figure 2) [17,18,19]. This process requires concerted efforts and depends on specific properties of each ADC component, released drug ideally also has a bystander effect.

### 2.2. Target and Antibody Selection for ADC

First, the selection of tumor-associated targets is critical for the success of ADCs as clinical anticancer therapeutics. Ideally, the target antigens should be expressed abundantly and uniformly on the surface of tumor cells and be absent in healthy tissues and organs. However, such ideal antigens are extremely rare, because an unrecognized antigen would trigger the immune response and lead to quick clearance [20]. In many cases, the optimal targets are expressed substantially higher in tumor cells than in normal cells. Such a difference can be identified by genomic, transcriptomic (microarray), and/or proteomic techniques [21]. It is worth noting that ADCs are efficacious over a wide range of antigen expression levels. For example, T-DM1 is an approved anti-human epidermal growth factor receptor 2 (HER2) ADC for metastatic breast cancer (MBC) and targets very high copy numbers of HER2 (in some cases, >3 million copies per cell) [22]. On the other hand, the ADC gemtuzumab ozogamicin targeting relatively low expression levels of CD33 (5000–10,000 receptors per cell) has shown potent activity in preclinical and clinical trials [23]. In addition, an optimal target must be localized on the tumor cell surface, can be recognized by its antibody, and has the ability of internalization upon ADC binding [24].

In addition to the selection of antigen, an optimal antibody is also important and has a significant impact on the therapeutic index, PK/PD profiles, and overall anticancer efficacy of an ADC. Antibodies can be classified into different types: IgA, IgG, IgD, IgE and IgM according to their biological properties and functional locations [25]. Based on the targets, currently used antibodies fall into the following categories, but are not limited to antibodies targeting leukocyte differentiation antigens (CD20, CD22, CD30, CD33, etc.), tyrosine kinase receptors (EGFR, EphA2, EphB2), glycoproteins (gpNMB, MUC16, PSMA, FAP, mesothelin, and TAG-72), sodium phosphate transporter (NaPi2b), oncoproteins (Cripto, ED-B, TMEFF2, CAIX), and hormone receptor (αv integrins) [26,27]. A suitable mAb in an ADC must retain high specificity and binding affinity to the target antigen on tumor cells, efficient receptor-mediated internalization, low immunogenicity, long circulation time, and antitumor activity against target cells after conjugating with the cytotoxic molecule through an appropriate linker [11,28]. First, the high affinity of a mAb to the target antigen is the most desirable characteristic, since it ensures good tumor localization and efficient internalization of ADCs into target cells. However, high affinity of the mAb may lead to fast binding to perivasculature with subsequent quick internalization of ADCs into the cells they first encounter, thus limiting further diffusion into the tumor [11]. Accordingly, the balance between penetration and retention ability of an antibody should be considered for a desired binding affinity [29]. Second, the molecular size is another important consideration. A full-length antibody having a long circulation half-life leading to a good chance of reaching the tumor site is thus favorable for an ADC. A long half-life, on the other hand, might be a risk for the degradation of an ADC and lead to nonspecific toxicity [11,29,30]. Preclinical studies have shown that smaller scaffold proteins or antibody fragments (single-chain variable fragments or Fab fragment) may achieve greater tumor penetration than might their parent antibodies [30,31]. Rapid clearance leading to a short half-life will still deter the clinical success of ADCs. Therefore, fine balances between molecular size and affinity and between tumor retention and penetration of an antibody will be critical for the success of an ADC [15,32].

### 2.3. Conjugation Strategy of Payload

The chemistry involving the conjugation strategy of payload is another important consideration for designing and assessing ADCs with regard to their PK/PD profile, binding affinity, toxicity, and stability. The early approach for preparing ADCs involves random conjugation of a small-molecule drug to either amines on lysine residues or sulfhydryl groups on cysteine residues of the antibody. The conjugation forms a heterogeneous mixture with each subpopulation displaying different PK/PD and efficacy properties. In contrast, controlled site-specific conjugation has the potential to overcome the heterogeneity limitations and widen the therapeutic window of ADCs [33,34]. Initial controls of the heterogeneity involved selective reduction of the four intermolecular disulfide bonds within an IgG. However, such trials caused the drug-antibody ratio (DAR) distribution of ADCs to vary from 0 to 8. This diversity of ADCs not only resulted in great challenges for subsequent purification but also influenced the circulation half-life, efficacy, and even increased the potential of off-target toxicity. Moreover, the maleimide-based ADCs, wherein the payloads are attached via thioethers, are prone to lose the payload through thiol exchange in the blood, though this limitation can be overcome by using cysteine bridging dibromomaleimides, *bis*-sulfone, and dibromopyridazinediones [35,36,37]. Another modification, using cysteine bridging bifunctional groups is to reduce the maximum number of cytotoxic drugs attached to a mAb from eight to four. Recently, a variety of approaches have been developed to obtain site-specific conjugations where sites of attachment of the cytotoxic agent to the antibody are precisely defined. These technologies are as follows: (a) applying engineered cysteine (such as THIOMAB enabling site-specific conjugation on only the heavy chain of the antibody) [38]; (b) introducing unnatural amino acids through mutagenesis to proteins and antibodies (such as incorporating p-acetylphenylalanine or selenocysteine into an IgG1) [39]; (c) using enzymatic and chemoenzymatic methods to generate site-specific conjugations (such as using engineered glycotransferases, transglutaminases, transpeptidase sortase, or formyl glycine generating enzymes to form the site-specific functionalization of antibodies) [40,41,42,43,44,45]; and (d) using tubulin-tag labeling strategies [46,47,48]. Details of these technologies are covered in previous reviews [19,34,49].

### 2.4. Linker Selection for ADC

Selecting a linker between the mAb and the cytotoxic agent is also important for ADC development. A linker must be stable enough in systemic circulation and be able to rapidly and efficiently release the cytotoxic agent upon internalization of the ADC within cancer cells [50]. According to the drug release mechanism, linkers for ADCs are generally categorized into cleavable linkers (acid-labile linkers, protease cleavable linkers, and disulfide linkers) and non-cleavable linkers (Table 1) [51]. The ADC with non-cleavable linker requires lysosomal degradation of the antibody for releasing the cytotoxic agent, while that with cleavable linker involves hydrolysis, enzymatic reaction, or reduction to selectively release the cytotoxic drug based on the physiological environment to which the ADC is exposed [50].

Three major types of cleavable linkers are often used in ADCs: acid-cleavable linkers, protease-cleavable linkers, and disulfide linkers. Acid-cleavable linkers such as hydrazine linkers are designed to be stable at a neutral pH of circulation but can be hydrolyzed in lysosomes with a low pH environment. Mylotarg, for example, was generated by conjugating calicheamicin with mAb against the CD33 antigen through an acid-cleavable hydrazine linker [52]. Protease-cleavable linkers are also used to keep ADCs intact in systemic circulation and allow easy release of the cytotoxic drugs from ADCs by lysosomal enzymes within cancer cells. For example, valine-citrulline (Val-Cit) and phenylalanine-lysine (Phe-Lys), which confer excellent stability and PK/PD profile to ADCs, have been used in many ADCs for preclinical and clinical evaluations. In addition, the ADCs with disulfide linkers take advantage of reduced glutathione with high intracellular concentrations to release free drug inside the cell. ADCs with reducible disulfide linkers generate uncharged metabolites that can diffuse into neighboring cells and elicit bystander killing, which is essential for killing heterogeneous tumors [51,53].

ADCs with cleavable linkers have broader efficacy and faster rates of activating and releasing cytotoxic drugs for most cell lines. In contrast, ADCs with non-cleavable linkers can possibly provide increased plasma stability, greater therapeutic window, and reduced off-target toxicity. To reduce aggregation and improve the solubility of some ADCs, hydrophilic linkers such as β-glucuronide linker, Sulfo-SPDB, and Mal-PEG4-NHS were investigated [54,55]. In short, each type of linker has advantages and disadvantages, and each can be modified to achieve a fine balance between target efficacies and undesired toxicities [54].

### 2.5. Payload Selection for ADC

The cytotoxic moiety (or payload) is the most important element of an ADC, and it should meet several requirements. First, the payload concentration in the target cells is often quite low, usually due to poor tumor localization and insufficient internalization. Therefore, a cytotoxic molecule with subnanomolar potency is required to exert optimal efficacy. In addition, the payload molecule should contain a functional group for conjugation or be capable of being chemically modified to generate an appropriate site whereby the original payload can be released from the ADC in tumor cells. Finally, the payload should be stable and soluble under physiological conditions. The payloads currently used for ADCs fall into the following four categories: tubulin inhibitor (e.g., maytansine analogs and auristatin analogs), DNA damaging agent (e.g., calicheamicin and pyrrolobenzodiazepine analogs), topoisomerase I inhibitor (e.g., SN-38), and RNA polymerase II inhibitor (e.g., α-amanitin).

## 3. Tubulin Inhibitors as Payloads of ADCs

**Figure 3:**
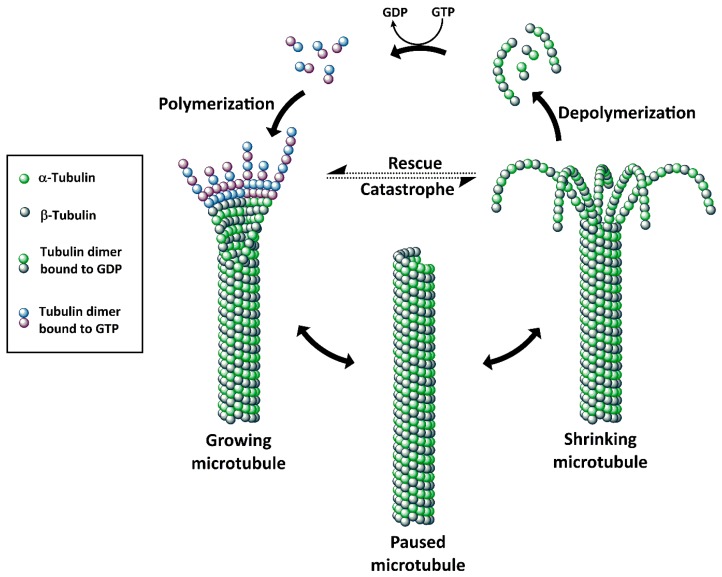
Structure, polymerization and depolymerization of microtubules.

**Figure 4:**
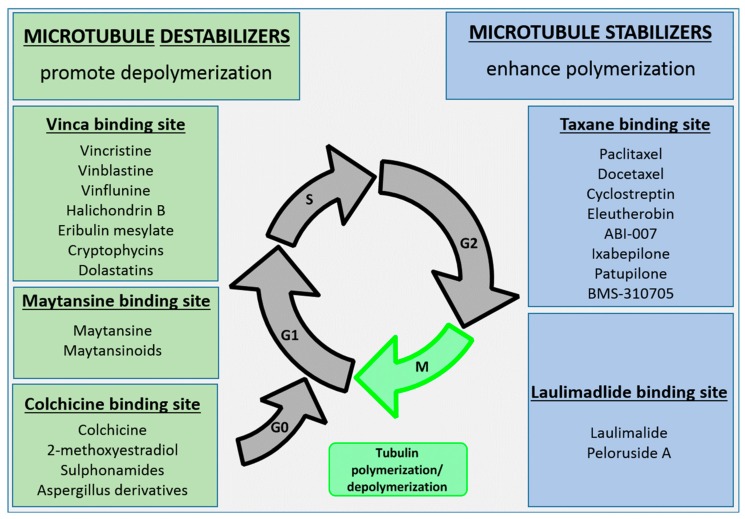
Classification of tubulin stabilizers and destabilizers arresting cell cycle at G_2_/M phase.

Microtubules, a dynamic assembly of α- and β-tubulin heterodimers (Figure 3) [56,57] are involved in many cellular processes such as cell structure maintenance, cell division, and intracellular transport. Disruption of microtubules induces cell cycle arrest in the G_2_/M phase, which makes microtubules an attractive target for drug discovery. To date, microtubules have five known binding sites: vinca alkaloid binding site, taxane binding site, colchicine binding site, maytansine binding site, and the laulimalide binding site. Microtubule/tubulin inhibitors can be classified into two major categories according to their mechanisms of action: agents promoting tubulin polymerization and stabilizing microtubule structures (e.g., paclitaxel, epothilones, discodermolide and taccalonolides), and agents inhibiting tubulin polymerization and destabilizing microtubule structures (such as maytansinoids, auristatins, vinblastine and vincristine) (Figure 4) [58,59]. The majority of the clinically used tubulin inhibitors are natural products and their synthetic derivatives, such as taxanes (e.g., paclitaxel) and vinca alkaloids (e.g., vinblastine) [60]. These earlier tubulin inhibitors, however, have intrinsic limitations including sensitivity to ATP-binding cassette transporter-mediated drug resistance and low cytotoxicity. These limitations resulted in studies leading to the discovery of new generations of tubulin inhibitors with greater potency. Maytansine was one of the first compounds with picomolar IC_50_ values and higher potency (100- to 1000-fold) than, for example, doxorubicin and paclitaxel.

**Figure 5:**
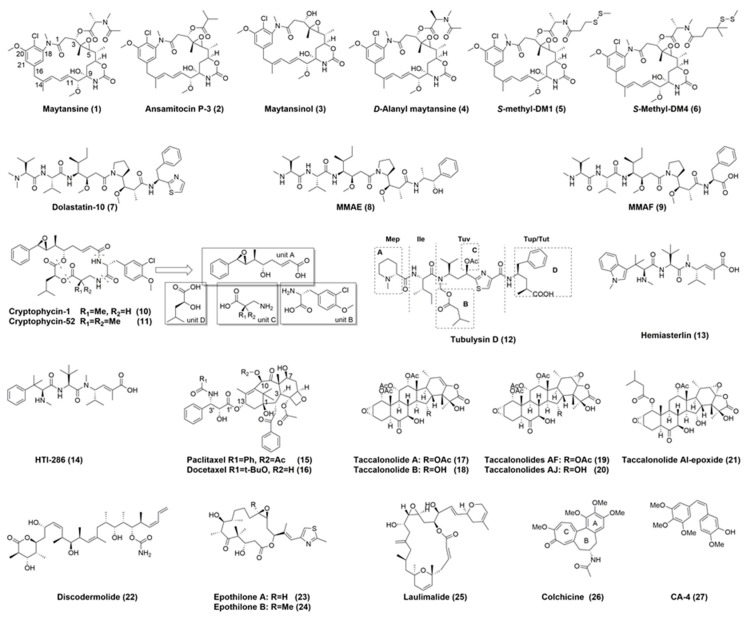
The chemical structures of tubulin inhibitors and their derivatives. Microtubule destabilizers: maytansine and its derivatives, dolastatin-10 and its derivatives, cryptopycin-1, cryptopycin-52, tubulysins D, hemiasterlin and its derivative (HTI-286), colchicine and CA4. Microtubule stabilizers: paclitaxel, discodermolide, taccalonolides A and B and their derivatives (taccalonolide AF and taccalonolide AJ), and taccalonolide AI-epoxide, laulimalide, and epothilones A and B.

However, in many cases, the poor therapeutic window and lack of tumor specificity led to failure of potent tubulin inhibitors alone as an anticancer agent. ADCs as targeted therapy might be a promising approach to address the limitations of single-agent therapy. Currently, tubulin inhibitors are one of the most validated payloads in ADCs and have been extensively investigated. There are two approved ADCs on the market today: T-DM1 and SGN-35, which use tubulin inhibitors maytansine-derivative DM1 (tubulin inhibitor targeting maytansine site) and dolastatin-derivative MMAE (tubulin inhibitor targeting vinca alkaloid site) as warheads, respectively. Tubulin inhibitors as ADC payloads have achieved some commercial successes. However, these ADCs are still far from the concept of ideal targeted therapy. Payloads which are active against drug-resistant tumors and have higher potency and better solubility, stability, tolerability, and therapeutic indices than current payloads, will be beneficial for the success of the next generation of ADCs [10]. More recently, some potent tubulin inhibitors suitable for payloads of ADCs were discovered and advanced to clinical phase evaluation. These new potential tubulin/microtubule agents for ADC payloads may overcome the current limitations of anticancer therapies. This section highlights both representative tubulin inhibitors (Figure 5) that have already been used in ADCs and novel tubulin inhibitors that are too toxic to be used as a single agent but are promising candidates for ADC payloads.

### 3.1. Maytansinoids as ADC Payloads

**Scheme 1:**
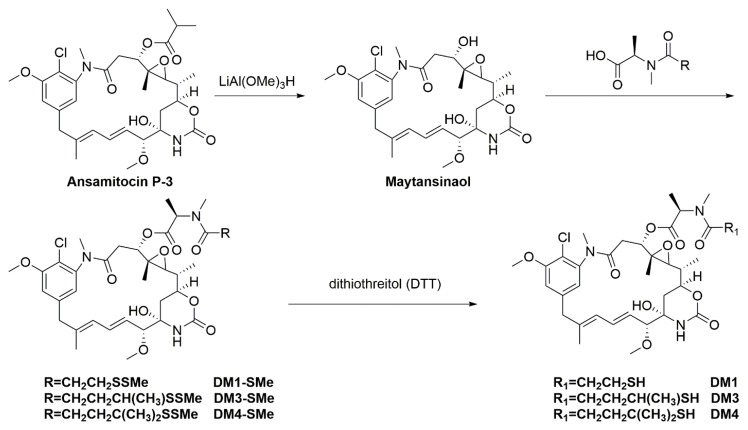
Semi-synthesis of DM1, DM3 and DM4 from ansamitocin P-3.

Maytansinoids are anti-mitotic tubulin inhibitors derived from maytansine (**1**) (Figure 5), a benzoansamacrolide that was initially isolated from an alcoholic extract in the bark of the African shrubs *Maytenus serrata* and *Maytenus buchananii* in 1972 [61]. Maytansine and maytansinoids bind to the maytansine site, resulting in the suppression of microtubule dynamics and causes cell cycle arrest in the G_2_/M phase. Maytansine showed potent anticancer activity in the picomolar range against most of the tested cell lines, and it was 100- to 1000-times more potent than were clinically used anticancer drugs in vitro [62]. Maytansine and its analogs were extensively evaluated in Phase I and Phase II clinical trials, with a dose limitation of 1–2 mg/m^2^. The dose-limiting toxicities include neurotoxicity, gastrointestinal toxicity, weakness, nausea, vomiting, and diarrhea. The poor therapeutic window and lack of tumor specificity led to the ultimate termination of the investigations on maytansine alone as an anticancer agent [63]. Due to the structural complexity including numerous stereocenters, the total synthesis of maytansine and its analogues is cost prohibitive even if it were achievable [64]. Maytansine is naturally generated from ansamitocins which were obtained from fermenting microorganism *Actinosynnema pretiosum*. Through a semi-synthesis strategy, a series of maytansine analogs (DM1, DM3 and DM4) bearing disulfide or thiol groups, which allow covalent linkage with mAbs, were obtained in two steps. The precursor, ansamitocin P-3 (**2**), was reduced by lithium trimethoxyaluminum hydride to give maytansinol (**3**). It was further esterified in the presence of dicyclohexylcarbodiimide and a Lewis acid (zinc chloride), to give maytansinoid disulfides, which were subsequently reduced by dithiothreitol (DTT) to yield DM1, DM3 and DM4 (Scheme 1) [65,66].

The structure-activity relationship (SAR) studies on maytansine showed that the C4–C5 epoxide moiety, the carbinolamide at C9, and double bonds at C11 and C13 are all required for optimal activity. The presence of the ester side chain (*N*-acyl-*N*-methyl-l-alanyl) at C3 is essential for activity and their corresponding l-epimers is about 100-fold more potent than the unnatural *N*-methyl-d-alanyl moiety (**4**). The side chain, however, can be modified to afford a series of maytansinoids bearing disulfide or thiol groups that do not result in the loss of activity [66,67].

**Scheme 2:**
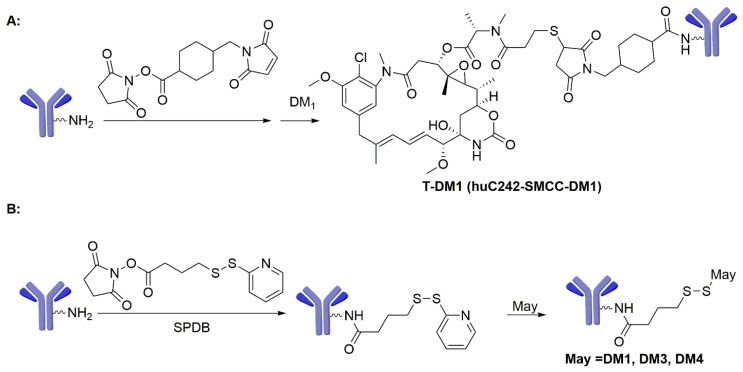
(**A**) conjugation of DM1 to antibodies by maleimide linker to produce antibody-SMCC-DM1 ADCs. SMCC: *N*-succinimidyl-4-(*N*-maleimidomethyl)cyclohexane-1-carboxylate; (**B**) conjugation of maytansinoids (May: DM1, DM3 and DM4) to antibodies by disulfide linker to produce antibody–SPDB–May ADCs. SPDB: *N*-succinimidyl-4-(2-pyridyldithio)butanoate.

Maytansinoids with potent cytotoxic activity are attractive candidates for ADC payloads. These maytansinoids may be linked to antibodies via a non-cleavable linker or a cleavable linker. The T-DM1 developed by Roche was prepared by reacting the antibody with succinimidyl 4-(*N*-maleimidomethyl)cyclohexane-1-carboxylate (SMCC), followed by reaction of the modified antibody with the thiol-containing maytansinoid, which demonstrates increased plasma stability, greater therapeutic window, and reduced off-target toxicity compared to that with cleavable linkers (Scheme 2A) [68]. It was approved by the FDA to treat metastatic breast cancer, which was previously treated with ado-trastuzumab and a taxane. The maytansinoid ADC with reducible-linkage cleavage, however, is operated by different mechanisms in dissimilar physiological environments (i.e., the persistent higher concentration of glutathione and disulfide isomerase enzymes in tumor tissues vs. the intracellular compartment). Representative maytansinoid ADCs based on disulfide linker are prepared through lysine residues from the antibody that can react with *N*-succinimidyl-4-(2-pyridyldithio)butanoate (SPDB) linker to give a modified antibody that contains a reactive disulfide. A thiol-bearing maytansinoid is then added by replacing 2-thiopyridine to give the maytansinoid conjugates (Scheme 2B) [69,70].

**Scheme 3:**
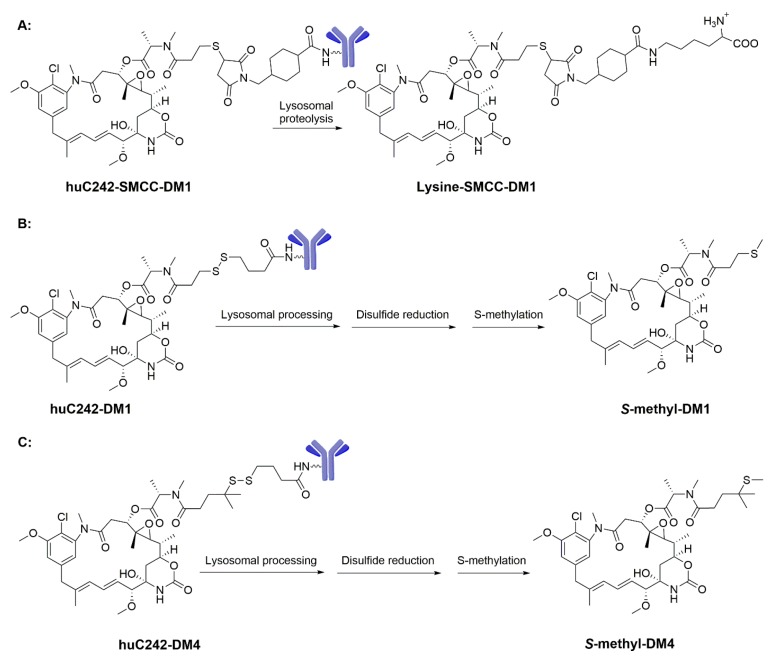
(**A**) antibody-SMCC-DM1 generates metabolite Lys-SMCC-DM1. SMCC: *N*-succinimidyl-4-(*N*-maleimidomethyl)cyclohexane-1-carboxylate; (**B**) metabolism of antibody-SPDB-DM4. SPDB: *N*-succinimidyl-4-(2-pyridyldithio)butanoate; (**C**) metabolism of antibody-SPDB-DM4.

**Scheme 4:**
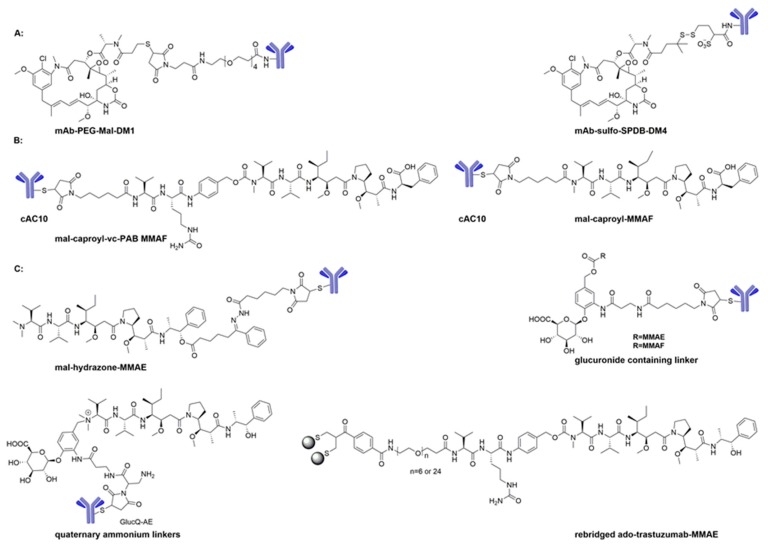
(**A**) the chemical structure of mAb-PEG-Mal-DM1 and mAb-sulfo-SPDB-DM4. PEG4: hydrophilic tetraethylene glycol, sulfo-PDB: sulfo *N*-succinimidyl-4-(2-pyridyldithio)butanoate; (**B**) the tubulin inhibitor MMAF-based ADCs; (**C**) the hydrazone functionalities, β-glucuronide containing linkers, quaternary ammonium linkers or rebridged ado-trastuzumab-based ADCs using MMAE or MMAF as payload.

The in vivo stability of the maytansinoid ADCs with two disulfide-linkages (huC242-DM1 and huC242-DM4) and a thioether-linked conjugate (huC242-SMCC-DM1) was proved through the latter’s exceptional half-life of 134 h in mice, a half-life superior to that of huC242–DM4 and huC242–DM1 (102 h and 47 h, respectively) [70,71]. The in vitro cytotoxicity studies showed that all of these conjugates were highly potent with IC_50_ values ranging from 3.5 to 15 pM. Erikson and co-workers [70,72] evaluated the metabolism, and indicated that the sole metabolite of the thioether-linked huC242-SMCC-DM1 was lysine-SMCC-DM1 (Scheme 3A), while the conjugates of huC242-DM1 and huC242-DM4 with disulfide linkers produced DM1 and *S*-methyl-DM1 (**5**) (Scheme 3B). Likewise, the disulfide-linked DM4 conjugates generated DM4 and *S*-methyl-DM4 (**6**) (Scheme 3C). These uncharged metabolites, *S*-methyl-DM1 and *S*-methyl-DM4, are able to efflux from the cell, diffuse into bystander cells, and induce cell death [53,70,72,73,74,75]. This may explain why ADCs with cleavable linkers have a broader efficacy for most cell lines. Many maytansinoid ADCs are substrates of the multidrug transporter MDR1 (also known as P-glycoprotein or P-gp), so several novel hydrophilic linkers (Sulfo-SPDB and Mal-PEG4-NHS) were developed to evade MDR1-mediated drug resistance (Scheme 4A) [76,77].

### 3.2. Auristatins as ADC Payloads

The auristatins are derived from the natural product dolastatin-10 (**7**) (Figure 5), which is isolated from the sea hare *Dolabella auricularia* found in the Indian Ocean and the coastal waters of Japan in an extremely low abundance [78]. Dolastatin-10 and its analogs inhibit tubulin-dependent GTP binding and block the binding of vinca alkaloids to tubulin in a noncompetitive manner [78,79]. Dolastatin-10 has demonstrated a wide spectrum of anticancer activity against a multitude of lymphomas, leukemia, and solid tumors with initial studies demonstrating IC_50_ values in the subnanomolar range. However, dolastatin-10 failed in Phase I and Phase II trials for non-small cell lung, melanoma, colorectal, ovarian, prostate, breast, and pancreatobiliary tumors as a single agent due to its high cytotoxic activity and narrow therapeutic window [80,81].

**Scheme 5:**
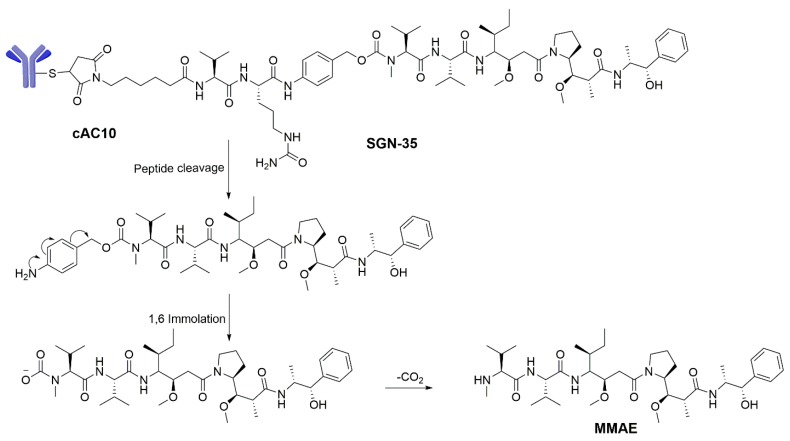
Metabolism and self-immolation of mc-Val-Cit-PABC-MMAE. mc: maleimidocaproyl. Val: valine. Cit: citrulline. MMAE: monomethylauristatin E.

To achieve appropriate cytotoxic ADC payloads, monomethyl auristatin-E (MMAE, **8**) and monomethyl auristatin-F (MMAF, **9**) were designed based on the structure of auristatin. Both MMAE and MMAF showed no degradation in plasma, in human liver lysosomal environment, or by the action of proteases. As free toxins, the cytotoxicities of MMAE and MMAF are less potent than that of dolastatin 10 in lymphoma cells in vitro. The ADC conjugating MMAE or MMAF with cAC10, however, showed similar cytotoxicity against CD30^+^ lymphoma cells with dolastatin-10 [15,82,83,84]. In addition, MMAE and MMAF are fully synthetic drugs, which are relatively easy to modify when optimizing their physical properties and characteristics. Likewise, MMAE and MMAF are unique linear pentapeptides containing unnatural amino acids with slight modifications in the *N*- or *C*-position, and retain their potency in the form of the ADC payloads. In vitro studies concluded that MMAF was less cytotoxic than was MMAE, because the charged carboxylic acid group hinders its ability to diffuse into the cells [71,83]. Different linking strategies including acid-labile or proteolytically cleavable linkers as well as non-cleavable linkers have been evaluated in auristatin ADCs. The proteolytically cleavable linkers are introduced to keep ADCs stable in systemic circulation and allow their easy release of the cytotoxic drugs by specific intracellular proteases such as cathepsin B [85]. The ADC SGN-35 was prepared by conjugating MMAE to a CD30-specific mAb via a protease-cleavable dipeptide linker, valine–citrulline (val-cit) as stated above [17,86,87,88,89]. The val-cit peptide can be hydrolyzed through lysosomal proteases, then the paraaminobenzyl (PAB)-MMAE subsequently undergoes self-immolation to release the original MMAE (Scheme 5) [85]. This is one of the ADCs on the market approved by the FDA in August 2011.

ADCs have also been designed to conjugate to cAC10 with mal-caproyl-vc-PAB MMAF and mal-caproyl-MMAF to give a protease-cleavable linkage and a non-cleavable linkage, respectively (Scheme 4B) [90,91]. Initial studies showed that the maximum tolerated dose (MTD) with non-cleavable linkage is higher than that with cleavable linkage. However, in some cases, the cytotoxicity of MMAF ADCs with cleavable linkage is more potent than that with non-cleavable linkage.

The acid-labile cleavable linkers, such as hydrazone functionalities, β-glucuronide containing linkers, or quaternary ammonium linkers have also been used for several auristatin ADCs for PK profile evaluation (Scheme 4C) [75,92,93]. To date, these compounds have not advanced to clinical trials. Most recently, a novel technology using cysteine rebridged ado-trastuzumab-MMAE to produce a homogeneous and stable conjugate with a DAR of 4 was investigated, and the conjugate was found to be superior in efficacy to T-DM1, as evaluated in a low HER2 expressing JIMT-1 xenograft model (Scheme 4C) [94].

### 3.3. Cryptophycins as ADC Payloads

The cryptophycins are a family of 16-membered cyclic depsipeptides, first isolated from terrestrial blue-green algae in 1990 [95]. They are potent tubulin-binding antimitotic agents. The most abundant component, cryptophycin-1 (**10**) (Figure 5), has demonstrated excellent activity against a broad spectrum of solid tumors with IC_50_ values in the picomolar range [96]. Significantly, it was not an effective substrate for the P-gp in multiple-drug resistant (MDR) cancer cell lines [97]. However, the intrinsically low bioavailability of cryptophycin-1 caused by its instability and solubility rendered it inefficacious in vivo. Chemists continued to explore and modify the scaffold, leading to the synthesis of hundreds of analogs [98].

The SAR can be summarized as follows: for unit A, the β-configuration of benzylic epoxide is essential and more potent than are the α-configured epoxides. The opening of the epoxide in the case of chlorohydrins also showed potent cytotoxicity. A series of *para*-substituted phenyls, such as hydroxyl, amino, carboxyl, ester, and amide derivatives, were designed, synthesized, and were proven to be more potent than *ortho*- or *meta*-substituted analogs. More importantly, the nature of the *para*-substitution can be modified as a conjugating site for the preparation of cryptophycin ADCs. Sanofi filed a patent based on the analogs described above, and they demonstrated effective antitumor activity with potentially improved physical, chemical, and biological profiles [99,100,101,102,103]. For unit B, the retention of d-tyrosine and benzene ring bearing *para*-methoxy or *ortho*-hydroxy is necessary for the activity. Studies showed that the B segment bearing the 5-fluorine substituted benzene ring lost bioactivity [99,104]. The C fragment structure-activity studies have focused on the C6 position, and it has been determined that the introduction of a bulky substituent such as a benzyl or isopropyl group resulted in a considerable reduction of bioactivity, while geminal methyl groups or a cyclopropyl group in the C6 position enhanced anticancer activity. In addition, it lost most activity if the C fragment was transformed into an alpha-amino acid, and the 16-membered ring property is essential for bioactivity, though the replacement of the ester bond between the C and D units with an amide derivative did not influence cytotoxicity. Nahrwold and co-workers reported that the replacement of an amide bond between units B and C by a triazole ring via Cu(I)-mediated “click” cyclization led to a reported IC_50_ value of 3.2 nM against the MDR tumor cell line KB-V1. Norbert Sewald’s group introduced the side chain at C6 containing an allyl and azide, and the resulted analogs were highly active in the cytotoxicity assay using the human cervix carcinoma cell line KB-3-1 and its MDR subclone KB-V1 [100,105,106,107]. The unit D side chain shows only slight variations relative to activity. Norbert Sewald’s group first introduced a side chain containing an ester bond or free carboxylic acid group. The functionalization of unit D precursors still retained good activity and provided a new site for biological coupling [108,109,110].

Cryptophycin-52 (Cr-52, LY355703, **11**) was designed by Eli Lilly, and its reported potency is 40- to 400-fold greater than that of paclitaxel or vinca alkaloids, making it a promising clinical candidate [111,112,113,114]. The mechanism of action involves suppressing microtubule dynamics and arresting cells in the G_2_/M phase. However, the co-crystallization data between Cr-52 and tubulin has not yet been solved, and the details of the binding site remain elusive. The Cr-52 structure is an analogue to cryptophycin-1, containing two hydroxy acids (units A and D) and two amino acids (units B and C). Due to the steric hindrance of the geminal dimethyl group, it is more resistant against ester hydrolysis than are cryptophycin-1 derivatives [103]. After numerous preclinical studies, Eli Lilly advanced Cr-52 to clinical trials, with aims to define the MTD, recommended dose, pattern of toxicity, and pharmacokinetic profile. Two Phase I clinical trials were performed, and a dose of 1.5 mg/m^2^ was recommended for Phase II evaluation on a day 1 and 8 schedule every 21 days [111].

Despite these findings, the treatment effect for NSCLC was not achieved as expected. Twenty-six patients were enrolled, of whom 25 were evaluable for toxicity and response. There were no responders when the weekly dose was lowered from 1.5 mg/m^2^ to 1.125 mg/m^2^. Median survival was 4.1 months, and toxicity was predominantly neurologic in the form of peripheral neuropathy and constipation [113]. Another study was performed to determine the activity of LY355703 and to characterize its toxicity profile in patients with platinum-resistant advanced ovarian cancer, but only modest activity was observed in these patients [114].

**Scheme 6:**
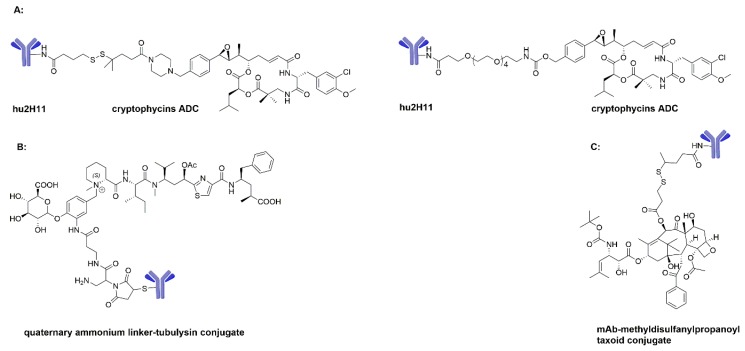
(**A**) the tubulin inhibitor cryptophycins-based ADCs; (**B**) the tubulin inhibitor tubulysins-based ADCs containing quaternary ammonium linkers; (**C**) the tubulin inhibitor taxoid-based ADCs.

Cr-52 failed in Phase II clinical studies due to the lack of efficacy and its high toxicity at the chosen doses. Although the cryptophycins were unable to advance to stand-alone agents, desirable characteristics including high potency, relative hydrophilicity, lack of P-gp susceptibility, and a common resistance mechanism make it an attractive ADC payload. Additionally, the *para*-substituted phenyl can be modified for the attachment of an antibody without losing activity. Sanofi designed a series of ADCs targeting EphA2 receptors by using hu2H11 antibodies coupling Cr-52. They were evaluated in MDA-MB-231 cell lines, and showed high bio-activity in early evaluation (Scheme 6A). However, further documented antitumor activity of Cr-52 ADCs has not been reported. Vishal A. Verma and coworkers used cryptophycins as potent payloads for ADCs through either a cleavable or non-cleavable linker and achieved satisfactory in vitro activity [115]. Further investigation may qualify Cr-52 as an acceptable candidate of ADCs for clinical evaluation.

### 3.4. Tubulysins as ADC Payloads

Tubulysins are cytotoxic natural products, originally isolated from myxobacterial cultures [116]. Structurally, tubulysins are tetrapeptides, composed of d-methylpipecolate (d-Mep), l-isoleucine (l-Ile), l-tubuvaline (l-Tuv), and l-tubuphenylalanine (l-Tup) residues. Among them, tubulysin D (**12**) (Figure 5) possesses the most potent activity with IC_50_ values between 0.01 and 10 nM, exceeding other tubulin modifiers including epothilones, vinblastine, and paclitaxel (Taxol) by 20- to 1000-fold. The mechanism of action of tubulysins resembles peptide antimitotics dolastatin-10, phomopsin A, and hemiasterlin, inducing the depletion of cell microtubules, arresting cells in the G_2_/M phase, and finally triggering cell apoptosis. Interestingly, microtubule depolymerization by tubulysins could not be prevented by preincubation with epothilone B and paclitaxel. In competition experiments, tubulysins interfered with vinblastine binding to tubulin in a non-competitive manner. In addition, tubulysins are less effective substrates of P-gp in MDR cancer cell lines [117].

Based on computer-aided drug design and biological electronic principles, a series of tubulysin derivatives with good biological activity have been synthesized. The clinical pharmacological data of these compounds have not yet been reported. Tubulysins are highly toxic tubulin-targeting agents with a narrow therapeutic window, making them interesting for target cancer therapy. Folate, ADCs, and polymeric tubulysin-peptide targeting nanoparticles enable tubulysins to achieve wider therapeutic indexes and improve their aqueous solubility [118,119,120,121].

Li et al. developed a biparatopic HER2-targeting ADC using tubulysin as payload, which showed superior antitumor activity over T-DM1 in tumor models representing various patient subpopulations [120]. The recently developed tubulysin ADCs with a novel quaternary ammonium linker have great stability profiles and potent anticancer activity based on in vitro and in vivo evaluation (Scheme 6B) [93]. Other than this, tubulysin ADCs are quite different from MMAE/MMAF and DM1/DM4 ADCs. The specific structural requirements of the tubulysin ADCs for optimal activity are still ambiguous at this stage. Further research on tubulysins as the ADC payloads may provide additional options for anticancer treatment.

SAR studies on tubulysins are shown in Figure 5: (a) the amine group of the Mep residue at the *N*-terminus is essential for activity; (b) the (*R*)-configuration of the *O*-acetate is preferred, and it influences tubulin assembly; (c) *N*,*O*-acetal groups can be eliminated without affecting cellular activity, and introducing a hydrophobic group improves bioactivity; and (d) the carboxylic acid group and the 3*R* configuration in the fragment D are essential for the activity [122,123].

### 3.5. Hemiasterlins as ADC Payloads

Hemiasterlin (**13**, Figure 5), a natural product, is a member of a small family of cytotoxic tri-peptides that were initially isolated from marine sponges [124]. Hemiasterlin and its synthetic analog HTI-286 (**14**) bind to the vinca binding site on the tubulin, inhibit the polymerization, and trigger mitotic arrest and subsequent apoptosis [125]. Studies demonstrated that hemiasterlin and its analogs stabilize the binding of colchicines to tubulin. They also inhibit the binding of vinblastine and dolastatin-10 to tubulin in noncompetitive and competitive manners, suggesting that hemiasterlin and dolastatin toxins share common microtubule protein binding sites, which is consistent with recently published structural biologic data [126,127].

Total synthesis of hemiasterlin and its analogs has been accomplished, and the SAR studies showed that the olefin at C2 and C3, the l-isopropyl and the l-methyl amino group at C4, and the geminal β,β-dimethyl group are all essential for activity. The *N*-methylindole could be replaced by phenyl and methyl groups, while still retaining potent antimitotic activity. HTI-286 and its analogue with a *para*-methoxyphenyl group are even more potent than hemiasterlin [128].

HTI-286 is less sensitive to P-gp protein than the current antimicrotubule agents such as paclitaxel, docetaxel, vinorelbine, and vinblastine. Phase I clinical trials in cancer patients dosed once every 21 days showed no objective responses. Side effects of HTI-286 include neutropenia, hair loss, and pain [129,130,131], leading to ultimate termination of phase II trials [130].

However, the preclinical evaluation of ADC micelle systems using hemiasterlins as payloads showed potent cytotoxicity against a broad range of tumor cells, reduced toxicity, and excellent therapeutic window. Hemiasterlin ADC is advanced as a potential clinical candidate for cancer therapy [132].

### 3.6. Paclitaxel and Docetaxel as ADC Payloads

Paclitaxel (Taxol, **15**, Figure 5), an amitotic inhibitor that binds and stabilizes microtubules, was first separated from the bark of the rare Pacific yew, later characterized by Wani et al. [133]. Paclitaxel and docetaxel (**16**) represent the taxane family of drugs, which showed remarkable efficacy against advanced solid tumors such as ovarian and breast cancer. In 1991, Holmes et al. reported the first clinical trial of paclitaxel to treat MBC with 250 mg/m^2^ over 24 h, and a 57% overall response was observed [134]. Despite these positive therapeutic features, paclitaxel seriously suffered from the lack of tumor specificity, MDR, poor aqueous solubility and dose-limiting toxicities including alopecia, nausea and vomiting, joint and muscle pain, peripheral neuropathy, and bone marrow suppression. To attenuate these side effects, many research groups have proposed that chemical coupling of mAbs with paclitaxel or its analogs would allow target delivery to tumor cells with increased efficacy and reduced side effects [135,136,137].

The first paclitaxel ADC with a cleavable ester linker was prepared by linking the paclitaxel C2′ hydroxyl group to glutaric anhydride to give 2′-glutarylpaclitaxel, whose carboxylic acid group then formed a peptide bond with the antibody. The resulted ADC showed better cytotoxic activity in vitro than paclitaxel or paclitaxel plus mAb, however, it showed only limited efficacy in vivo. In addition, Ojima et al. developed an EGFR-targeting ADC using a second-generation taxoid (C-10 methyl-disulfanylpropanoyl taxoid) as payload. This ADC showed remarkable target-specific antitumor activity in vivo (Scheme 6C) [136]. However, it remains elusive whether the effects are truly due to immunologically specific drug delivery or not. In short, the paclitaxel-based ADCs did not advance into clinical trials since paclitaxel and its analogs display insufficient cytotoxicity in many cell lines. It has thus been suggested that they are not suitable for ADCs. The development of next generation taxoids with IC_50_ of 10–100 pM will be the research direction using paclitaxel analogs as payloads.

The structure-activity relationship studies of paclitaxel showed that C-3’ with an alkyl or alkenyl group, C3’-N with a *tert*-butoxycarbonyl group, and acylation at C-10 are important to improve its activity against drug-resistant cancer cells. In addition, the benzoate at the C-2 can be replaced by alkenyl and alkyl groups, which provide nonaromatic second-generation toxoids [138].

### 3.7. Other Tubulin Inhibitors as Potential ADC Payloads

Other tubulin inhibitors (Figure 5) investigated include taccalonolide A or B (**17** or **18**), taccalonolide AF or AJ (**19** or **20**), taccalonolide AI-epoxide (**21**), discodermolide (**22**), epothilone A (**23**) and B (**24**), laulimalide (**25**), colchicine (**26**), CA-4 (**27**), and their respective synthetic derivatives. Although these compounds were not successful while being used alone as anticancer agents, they had the potential to be used as ADC payloads. Discodermolide remains the most potent natural promoter of tubulin assembly, is effective against paclitaxel-resistant cell lines, and also has synergistic effects with paclitaxel [139,140]. The epoxidation of taccalonolide yielded its analogs with strong cytotoxicity and unique interaction mechanisms (specifically, covalent bonding) to tubulin; thus, these are promising candidates for developing a new generation of irreversible tubulin inhibitor-based ADC drugs [141,142,143]. Epothilone, laulimalide, colchicine, and CA-4 are easy to be chemically modified, and they are not P-gp substrates and are active against P-gp related drug-resistance. As a result of being introduced to better conjugation groups (sites), these inhibitors could possibly be used as ADC payloads [144,145,146,147,148]. Future studies on tubulin inhibitor-based ADC payloads may help elucidate the drug resistance mechanisms and thus allow the design of drugs that are active against drug-resistant tumor cells but not P-gp substrates. In addition, all tubulin inhibitors so far target β-tubulin; discovering compounds with different tubulin inhibition mechanisms (such as α-tubulin inhibition) will open alternative avenues and provide a new class of payloads with different tolerability and therapeutic index from currently available ADCs [149]. Another direction may take advantage of hybrid payloads targeting different binding sites on tubulin simultaneously to overcome the intrinsic limitations of drug resistance and to improve the activity of payload drugs synergistically.

The tubulin inhibitor-based ADCs in preclinical and/or clinical trials and their corresponding status, therapeutic indications, targets, mAbs, linkers, and payloads are summarized in Table 2. Data were generated from the Thompson Pharma Partnering database and clinical trials.

## 4. Conclusions and Final Remarks

Encouraged by the in-depth understanding of ADCs for targeted therapy in oncology and the approvals of SGN-35 and T-DM1 by the FDA, ADCs are rapidly moving from empiricism to rational design. Nevertheless, ADCs are far from fulfilling the concept of a “magic bullet” specifically delivering cytotoxic drugs to tumor cells. Additionally, toxicity of ADCs to normal cells/tissues caused by undesired payload release needs to be considered. Studies have indicated that the delivery efficacy of ADCs into the target tumor cells was far less than 1% [150]. The limitations of ADCs must be addressed by future studies to propel this concept forward into relevant clinical regimens. Successful development of ADCs requires vast understanding of each ADC component, connecting each part with sufficient efficiency, expanding the therapeutic index, and improving the PK/PD profile. In addition, a deeper understanding of antigen targets, mechanisms of internalization, roles of novel linkers, executions of appropriate antibodies, and properties of the payload (such as cytotoxicity, specificity, solubility, and stability) are all essential criteria for the overall efficacy of ADCs. Advancement and implementation of site-specific conjugation technologies must be investigated and used to improve the safety and efficiency of ADCs as well. In short, the convergence of a thorough understanding of ADC-related technologies will be the driving force behind the design and development of novel ADCs, which will be promising tools for targeted cancer therapy in the future.

## Figures and Tables

**Table 1:** Summary of commonly used linkers for the development of ADCs.

Abbreviation	Chemical Structure	Linker Type
MHH	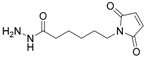	Chemical labile (acid labile) linker
DSDM	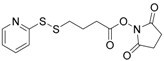	Disulfide-containing reducible linker
Sulfo-SPDB	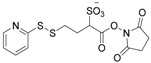	Disulfide-containing reducible linker
MC-VC-PABC	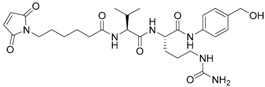	Enzymatically cleavable linker
SMCC	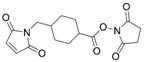	Non-cleavable bifunctional linker
Mal-PEG-NHS	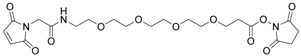	Non-cleavable spacer linker
GBC	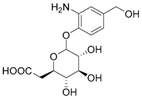	Enzyme-labile ß-Glucuronide linker

**Table 2:** Summary of the preclinical and clinically tested ADCs employing the maytansinoid, dolastatin, cryptophycin hemiasterlin, tubulysin and taxol as payloads.

ADC	Status	Therapeutic Area	Target	mAb	Linker	Payload
Trastuzumab emtansine (T-DM1)	Approved	Breast cancer	HER2	Trastuzumab	SMCC	DM1 ^a^
Lorvotuzumab mertansine (IMGN901)	Phase II	Hematologic-blood cancer, sarcoma, neuroblastoma, SCLC, MM	CD56	huN901	SPP	DM1
IMGN529	Phase II	NHL	CD37	K7153A	SMCC	DM1
IMGN289	Phase I	Squamous Cell carcinoma, NSCLC, solid tumors	EGFR	J2898A	SMCC	DM1
AMG 172	Phase I	RCC	CD27L	Anti-CD27L	MCC	DM1
AMG 595	Phase I	Glioma, AA	EFGRvIII	Anti-EGFRvIII	SMCC	DM1
MLN-2704	Discontinued	Prostate cancer	PSMA	MLN-591	SPP	DM1
Cantuzumab mertansine (HuC242-DM1)	Discontinued	NSCLC, pancreatic cancer, colorectal cancer, solid tumors	CanAg	huC242	SPP	DM1
Mirvetuximab soravtansine (IMGN853)	Phase II	Solid tumors, NSCLC, ovarian cancer	Folate receptor 1	M9346A	sSPDB	DM4 ^a^
Anetumab ravtansine (BAY94-9343)	Phase II	NSCLC, Mesothelioma, solid tumors	Mesothelin	Anti-mesothelin	SPDB	DM4
Coltuximab ravtansine (SAR3419)	Phase II	LBCL	CD19	huB4	SPDB	DM4
BT-062	Phase I/II	MM	CD138	nBT062	SPDB	DM4
SAR-428926	Phase I	Solid tumors	LAMP-1	853K3	SPDB	DM4
SAR-566658	Phase I	Solid tumors	CA6	huDS6	SPDB	DM4
IMGN388	Discontinued	Solid tumors	Integrin α_v_	Anti-integrin α_v_	SPDB	DM4
BIIB015	Discontinued	Solid tumors	Crypto	Anti-cripto	SPDB	DM4
AVE-9633	Discontinued	AML	CD33	huMy9-6	Undisclosed	DM4
Cantuzumab ravtansine (huC242-DM4)	Discontinued	Gastric cancer, pancreatic cancer	CanAg	huC242	SPDB	DM4
Adcetris (SGN-35)	Approved	HL, ALCL	CD30	cAC10 (SGN-30)	VC	MMAE ^b^
Glembatumumabvedotin (CDX-011)	Phase II	Breast cancer, melanoma, osteosarcoma, NSCLC	GPNMB	CR-011	VC	MMAE
Pinatuzumab vedotin (DCDT2980S)	Phase II	NHL, DLBCL	CD22	Anti-CD22	VC	MMAE
Polatuzumab vedotin (DCDS4501A)	Phase II	NHL, DLBCL, CLL	CD79b	Anti-CD79b	VC	MMAE
Lifastuzumab vedotin (DNIB0600A)	Phase II	Ovarian cancer, NSCLC	NaPi2b	Anti-NaPi2b	VC	MMAE
PSMA ADC	Phase II	Prostate cancer	PSMA	Anti-PSMA	VC	MMAE
DMOT-4039A	Phase I	Pancreatic cancer, ovarian cancer	MSLN	MMOT-0530A	VC	MMAE
AGS-22M6E (ASG-22CE)	Phase I	Genitourinary cancer, solid tumors	Nectin-4	Anti-Nectin-4	VC	MMAE
SGN-LIV1A	Phase I	Breast cancer	LIV1	Anti-LIV1	VC	MMAE
HuMax-TF-ADC	Phase I	Solid tumors	tissue factor	TF-011	VC	MMAE
AGS-15E	Phase I	urothelial	SLITRK6	AGS15	VC	MMAE
DLYE-5953A	Phase I	breast cancer, NSCLC, solid tumors	LY6E	Anti-Ly6E	VC	MMAE
DEDN-6526A	Phase I	Melanoma	EDNRB	Anti-EDNRB	VC	MMAE
AGS-67E	Phase I	Hematologic-blood cancer, AML	CD37	Anti-CD37	VC	MMAE
MLN-0264	Discontinued	Pancreatic cancer, gastric cancer	GCC	5F9	VC	MMAE
ASG-5ME	Discontinued	Pancreas cancer, Prostate cancer, gastric cancer	AGS-5	Anti-AGS-5	VC	MMAE
Bay 79-4620	Discontinued	Solid tumors	CA9	3ee9	VC	MMAE
Sofituzumab vedotin (RG-7458)	Discontinued	Ovarian cancer, pancreatic cancer	MUC16	Anti-MUC16	VC	MMAE
Vandortuzumab vedotin (DSTP-3086S)	Discontinued	Prostate cancer	STEAP1	Anti-STEAP1	VC	MMAE
Denintuzumab mafodotin (SGN-CD19A)	Phase II	DLBL, follicular lymphoma	CD19	Anti-CD19	MC	MMAF ^b^
AGS-16M8F	Phase II	RCC	ENPP3	Anti-AGS-16	MC	MMAF
Depatuxizumab mafodotin (ABT-414)	Phase II	Glioblastoma multiforme, NSCLC, brain cancer, solid tumors	EGFRvIII	ABT-806	MC	MMAF
ARX-788	Phase I	Breast cancer	Her2	Anti-Her2	PEG4	MMAF
J6M0-mcMMAF (GSK-2857916)	Phase I	MM	BCMA	J6M0	MC	MMAF
Vorsetuzumab mafodotin (SGN-75)	Discontinued	NHL, RCC	CD70	HIF6	MC	MMAF
PF-06263507	Discontinued	Solid tumors	5T4	Anti-5T4	MC	MMAF
MEDI-547	Discontinued	Ovarian cancer, solid tumors	EphA2	1C1	MC	MMAF
Hu2H11-SPDB-Ex1	Preclinical	Breast cancer, colon cancer,	EphA2	hu2H11	SPDB	Cryptophycin analogue ^c^
Hu2H11-SPDB-Ex2	Preclinical	Breast cancer, colon cancer,	EphA2	hu2H11	SPDB	Cryptophycin analogue
Hu2H11-SPDB-Ex5	Preclinical	Breast cancer, colon cancer,	EphA2	hu2H11	SPDB	Cryptophycin analogue
Hu2H11-SPDB-Ex6	Preclinical	Breast cancer, colon cancer,	EphA2	hu2H11	SPDB	Cryptophycin analogue
Hu2H11-Ex17	Preclinical	Breast cancer, colon cancer,	EphA2	hu2H11	Undisclosed	Cryptophycin analogue
Hu2H11-Ex18	Preclinical	Breast cancer, colon cancer,	EphA2	hu2H11	Undisclosed	Cryptophycin analogue
Hu2H11-Ex19	Preclinical	Breast cancer, colon cancer,	EphA2	hu2H11	Undisclosed	Cryptophycin analogue
VDC-886	Preclinical	Prostate cancer, breast cancer, NHL	pl-CS	DBL1-ID2a	Undisclosed	KT886 ^d^
NC-6201	Preclinical	Breast cancer, pancreatic cancer, colon cancer	EGFR	NCAB001	PEG-poly(glutamic acid benzyl ester)	E7974 ^d^
MEDI-4276 (AZ13599185-Trastuzumab)	Phase I	Breast cancer, gastric cancer	HER2	39S	MC	AZ13599185 ^e^
^131^I-TUB-OH-Trastuzumab	Preclinical	Breast cancer	HER2	Trastuzumab	-	TUB-OH ^e^
^131^I-TUB-OMOM-Trastuzumab	Preclinical	Breast cancer	HER2	Trastuzumab	-	TUB-OMOM ^e^
DX-109	Preclinical	Breast cancer	HER2	anti-HER2	Thioether	Tub-001 ^e^
αCD30–glucQ-Tub	Preclinical	HL, ALCL	CD30	AC10	Quaternary Ammonium	Tubulysin M ^e^
α-CEA-680-PTX	Preclinical	Pancreatic cancer	CEA	Anti-CEA	Hemisuccinamide-DyLight™ 680 − 4 × PEG	Paclitaxel ^f^

Note: payload based on ^a^ maytansinoid; ^b^ dolastatin; ^c^ cryptophycin; ^d^ hemiasterlin; ^e^ tubulysin and ^f^ taxol. The reasons for discontinued ADCs as follows: MLN-2704: sufficient therapeutic window was not achievable; Cantuzumab mertansine (HuC242-DM1): unforeseen or unacceptable toxicities; IMGN388: strategic reasons; AVE-9633: absence of evidence of clinical activity; Cantuzumab ravtansine (huC242-DM4): ocular toxicities; MLN-0264: lack of efficacy; ASG-5ME: strategic reasons; Bay 79-4620: safety reasons; some others: unknown.

## Abbreviations

ADCs	Antibody-drug conjugates
ALCL	Anaplastic large-cell lymphoma
AML	Acute myelogenous leukemia
BCMA	B-cell maturation antigen
CA9	Carbonic anhydrase IX
CEA	Carcinoembryonic antigen
CLL	Chronic lymphocytic leukemia
DAR	Drug-to-antibody ratio
DLBCL	Diffuse large B cell lymphoma
DTT	Dithiothreitol
EDNRB	Endothelin receptor type B
EGFR	Epidermal growth factor receptor
ENPP3	Ectonucleotide pyrophosphatase/phosphodiesterase family member 3
EphA2	Ephrin type-A receptor 2
EphB2	Ephrin type-B receptor 2
FDA	Food and Drug Administration
GCC	Guanyl cyclase C
GPNMB	Transmembrane glycoprotein NMB
GTP	Guanosine triphosphate
HER2	Human epidermal growth factor receptor 2
HL	Hodgkin’s lymphoma
IgG	Immunoglobulin G
LBCL	Large B-cell lymphoma
LY6E	Lymphocyte antigen 6 complex
mAb	Monoclonal antibody
MBC	Metastatic breast cancer
MC	Maleimidocaproyl
MCC	4-(*N*-Maleimidomethyl)cyclohexane-1-carboxylate
MDR	Multiple-drug resistant
MDR1	Multidrug resistance protein 1
MM	Multiple myeloma
MMAE	Monomethyl auristatin E
MMAF	Monomethyl auristatin F
MTD	Maximum tolerated dose
MUC16	Mucin 16
NHL	Non-Hodgkin’s lymphoma
NSCLC	Non-small cell lung cancer
PEG	Polyethylene glycol
PK/PD	Pharmacokinetic/pharmacodynamics
PSMA	Prostate specific membrane antigen
RCC	Renal cell carcinoma
SAR	Structure-activity relationship
SCLC	Small-cell lung cancer
SLITRK6	SLIT and NTRK-like protein 6
SMCC	Succinimidyl 4-(*N*-maleimidomethyl) cyclohexane-1-carboxylate
SPDB	*N*-Succinimidyl-4-(2-pyridyldithio)butanoate
SPP	*N*-Succinimidyl 4-(2-pyridyldithio) pentanoate
sSPDB	Sulfo-SPDB
STEAP1	Six transmembrane epithelial Antigen of prostate 1
TAG-72	Tumor-associated glycoprotein-72
vc	Valine-citrulline
